# Bony Lesions in Pediatric Acute Leukemia: Pictorial Essay

**DOI:** 10.5812/iranjradiol.6765

**Published:** 2012-03-25

**Authors:** Makhtoom Shahnazi, Alireza Khatami, Bibishahin Shamsian, Bibimaryam Haerizadeh, Mastooreh Mehrafarin

**Affiliations:** 1 Department of Radiology, Loghman-e Hakim Hospital, Shahid Beheshti University of Medical Sciences, Tehran, Iran; 2 Department of Radiology, Mofid Children's Hospital, Shahid Beheshti University of Medical Sciences, Tehran, Iran; 3 Department of Pediatric Hematology-Oncology, Mofid Children's Hospital, Shahid Beheshti University of Medical Sciences, Tehran, Iran; 4 Department of Radiology, Shahid Beheshti University Affiliated Hospitals, Tehran, Iran; 5 Mofid Children's Hospital, Shahid Beheshti University of Medical Sciences, Tehran, Iran

**Keywords:** Radiography, Leukemia, Child

## Abstract

Acute leukemia is the most common malignancy in childhood, which mainly involves children less than 15 years of age. The growing skeleton is the main site of involvement in children. Leukemic cells proliferate within the massive red bone marrow in children. So besides the pallor, petechia, purpura and ecchymosis in the skin and mucosal surfaces, bone pain and other bony lesions are other manifestations of leukemia.

On the other hand, bony lesions are more prevalent in children than adults with no poor prognosis in comparison to patients without bone lesions. These bony lesions may precede other laboratory tests so familiarity with these presentations is very important for earlier diagnosis.

In this pictorial essay, we tried to gather the most common bony lesions that may be seen in acute leukemia in different cases admitted to our hospital with general malaise and localized tenderness and discomfort leading us to perform plain X-ray for further evaluation. Finding these bony lesions helps clinicians to reach the diagnosis quickly. These findings include metaphyseal lucent band and erosion, periosteal reaction, small lucent bone lesion and permeative appearance, reduced bone density and collapsed vertebra.

## 1. Introduction

**Figure 1:**
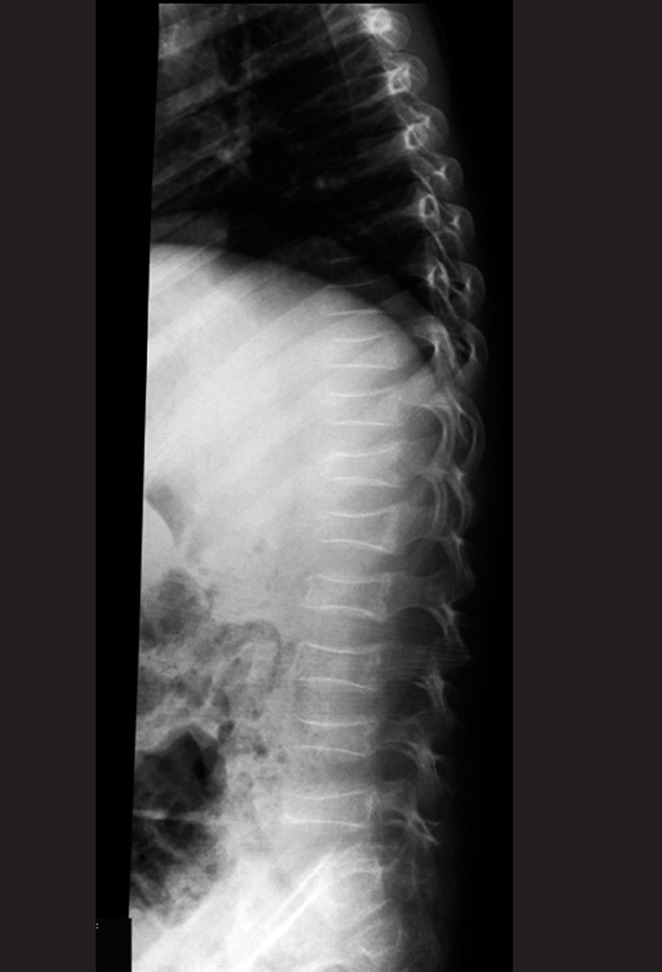
A 4-year-old girl with ALL presenting with back pain and pancytopenia Lateral thoracolumbar X-ray reveals multiple collapsed vertebrae.

**Figure 2:**
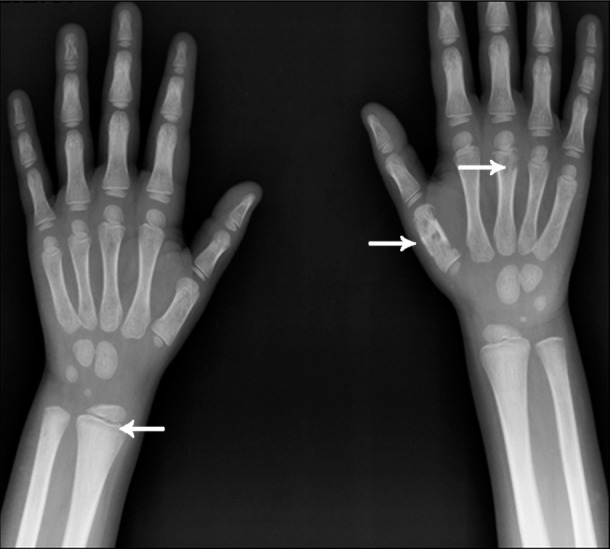
A 4-year-old girl with ALL presenting with pallor and wrist tenderness Wrist X-ray reveals metaphyseal lucent band (arrow head) in radial and ulnar metaphyses. Note the lucent bony lesions of the first and third metacarpal bone on the right side (arrows).

**Figure 3:**
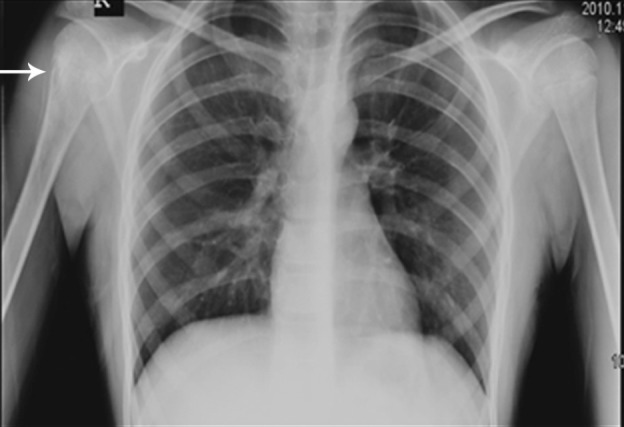
An 8-year-old boy with ALL presenting with cough, musculoskeletal pain and anemia CXR reveals permeative bone lesion in the right humerus (arrow).

**Figure 4:**
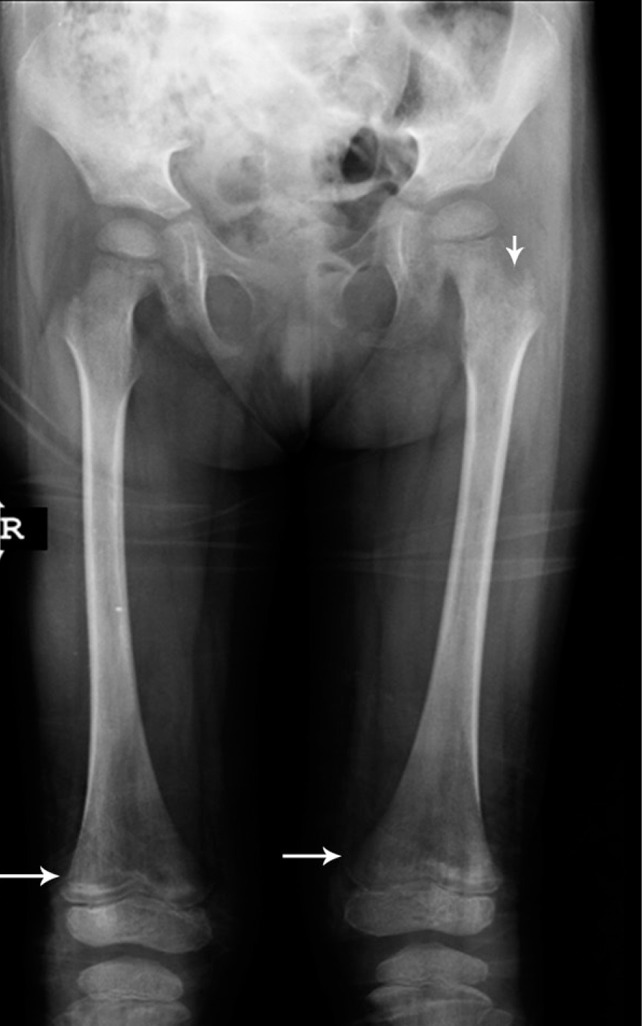
A 6-year-old girl with AML presenting with weakness, pallor and tenderness of lower extremities. Radiography reveals metaphyseal hypodensity on both femurs with sclerotic lesions (arrows). There are also lytic lesions in the left femoral neck (arrowhead).

**Figure 5:**
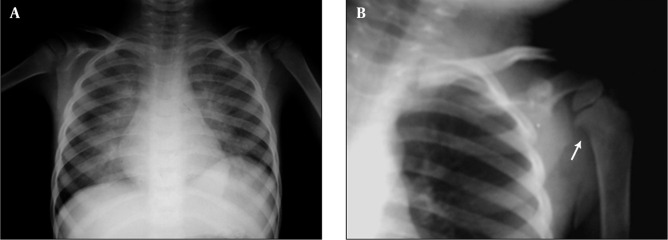
A 5-year-old boy with AML presenting with cough, fever and dyspnea A, CXR reveals bilateral parahilar alveolar infiltration in both lung fields. There is suspicious right humeral metaphyseal erosion; B, Magnification view reveals erosion more clearly with reduced adjacent bone density (arrow).

**Figure 6:**
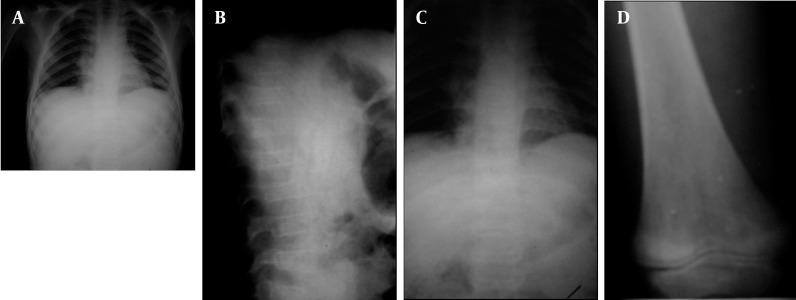
An 8-year-old boy with ALL presenting with anemia, fever, back pain and limping A, CXR reveals perihilar and retrocardiac infiltration. Abnormal shaped vertebra is suspicious.; B and C, Thoracolumbar AP and Lat X-Rays reveal multiple collapsed vertebrae; D, Knee X-xay reveals hypodensity around the knee joint with metaphyseal translucency.

**Figure 7:**
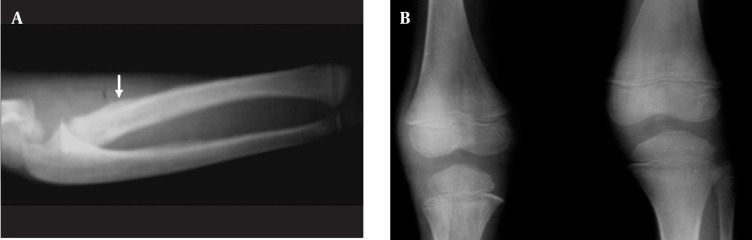
A 6-year-old girl with ALL presenting with pallor and right elbow pain A, Forearm X-Ray reveals periosteal reaction of radial proximal metaphysis with distal metaphyseal translucency(arrow);B, AP X-ray of knee joints reveals metaphyseal translucency in both distal femurs and proximal tibiae more prominent in the latter.

**Figure 8:**
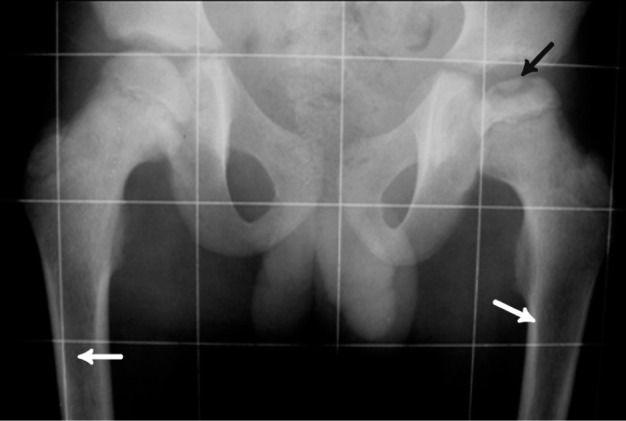
A 9-year-old boy with ALL presenting with groin pain and limping Pelvic X-Ray of this case reveals reduced height of femoral epiphysis with osteochondral fracture on the left side due to avascular necrosis (black arrow). Permeative lesion in both femoral proximal metaphyses are noted (white arrows).

**Figure 9:**
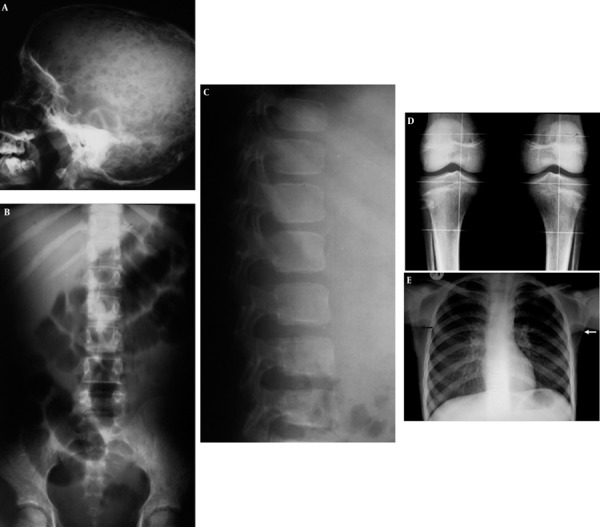
A 13-year-old girl with ALL presenting with pallor, fever, headache and bone pain A, Skull X-ray reveals multiple lytic lesions; B, Lumbosacral AP X-ray reveals permeative bony lesions in iliac bones with reduced vertebral height of L5; C, Lumbosacral lateral X-ray reveals reduced vertebral height of L5 with reduced bone density and lucent zone beneath the vertebral endplate; D, Knee X-ray reveals reduced bone density with permeative appearance; E, CXR reveals permeative bony lesions in bilateral scapular bones (arrows).

Acute leukemia is the most common malignancy in children that comprises about 41% of the malignancies in children younger than 15 years old [1]. It is the second most common malignancy in children under one-yearold [2][3]. Acute lymphocytic leukemia (ALL) is the most common malignancy under 15 years age. ALL is responsible for approximately 23% of all cancers and 76% of leukemia in this age group. However, only 20% of acute leukemia in adults is ALL [2][3]. The peak age of involvement is 2-6 years and boys are slightly more involved than girls. Chromosomal abnormalities such as Down, Bloom and Fanconi Syndrome and also ataxia telangiectasia are prone to this malignancy [1][4][5]. Although leukemia may present with pallor, petechia, ecchymosis in skin and mucus membranes, growing skeleton is an important site for proliferation of leukemic cells; therefore, during the course of disease, tenderness and multiple areas of bone destruction and repair due to infiltration of leukemic cells in the bone marrow may be seen [6][7]. More common radiographic findings which have been reported in the literature are generalized reduced bone density, metaphyseal lucent band, lytic bone lesions, metaphyseal cortical bone erosions, collapsed vertebra and widening of sutures and periosteal reactions [6][7] which have been seen in our cases with variable frequencies (Figure 1, 2, 3, 4, 5, 6, 7, 8, 9, & 10).

Due to widespread red bone marrow in childhood, more than 50% of children with leukemia reveal skeletal abnormalities; however, this is less than 10% in adults [7][8]. In addition, bone involvement had no worse prognosis in comparison to cases without bone involvement [6]. Although diagnosis of the disease is made by bone marrow aspiration, bony lesions may precede clinical findings [9]. Knowledge of radiographic and orthopedic appearances of leukemia is important in the diagnosis, supportive treatment and follow up of patients in order to improve their survival [10].

**Figure 10:**
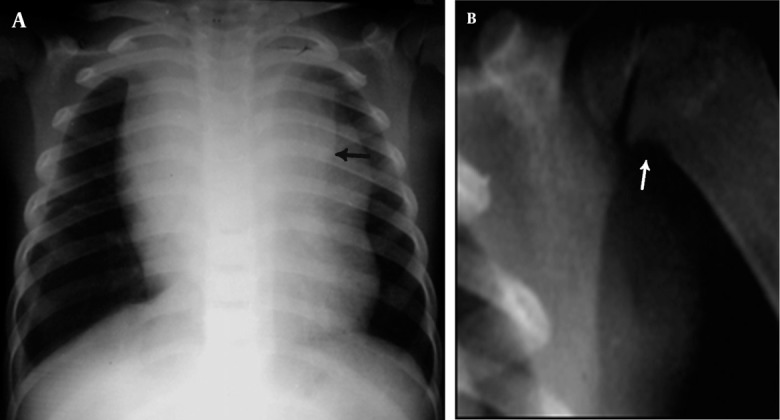
A 2-year-old girl with ALL presenting with fever and dyspnea A, CXR reveals anterior mediastinal widening with double contour thymus (arrow) and right pleural effusion; B, Magnification view reveals metaphyseal erosion in proximal medial humeral metaphysis (arrow).

## 2. Case Presentation

We have presented different cases with acute lymphoblastic and myeloblastic leukemia.

### 2.1. Case 1

A 4-year-old girl presented to the emergency department with severe back pain. CBC revealed pancytopenia and anemia. Back pain should always be considered pathologic in the pediatric age so the patient was sent to the radiology department for thoracoabdominal radiography. As can be seen, multiple dorsolumbar-collapsed vertebrae are detected (Figure 1). Bone marrow aspiration confirmed the diagnosis of ALL.

### 2.2. Case 2

Another 4-year-old girl was admitted to our hospital with a history of pallor and weakness. Wrist tenderness was obvious. The patient’s wrist x-ray revealed metaphyseal translucency that traverse radial and ulnar metaphyses bilaterally (Figure 2). There are also some lucent lesions in the first and third metacarpal bones. The patient had ALL.

### 2.3. Case 3

An 8-year-old boy admitted to the hospital clinic for cough and musculoskeletal pain. Primary evaluation as CBC, ESR and CXR was done. Hemoglobin was 8 mg/dL and ESR was 80mm. Petechia on the trunk was noticed. The CXR is depicted in Figure 3. Obviously right humeral bone permeative appearance with periosteal reaction is noticed. Otherwise reduced bone density in the visible bones is seen. This case was also a new ALL case.

### 2.4. Case 4

A 6-year-old girl was admitted to our hospital with weakness and muscle pain. She was pale and had lost her appetite. More tenderness in lower extremities was observed so she was referred to the radiology department for both femurs X-ray. The bone density was reduced. There are prominent hypodensities in distal femoral metaphyses and proximal tibial metaphyses with some area of sclerosis. Some lytic lesions exist in the left femoral neck. Otherwise the bone density is reduced (Figure 4). The patient was sent for bone marrow aspiration. The final diagnosis was acute myeloid leukemia (AML).

### 2.5. Case 5

A 5-year-old boy was admitted to our department with fever, breathlessness and cough. CXR was carried out and bilateral parahilar alveolar infiltration in both lung fields is seen. However, there was a suspicious right humeral metaphyseal erosion which could be seen in magnification view (Figure 5). Metaphyseal erosion is one of the presenting features of leukemia. The patient had AML with Pneumocystis carinii infection of the lung.

### 2.6. Case 6

An 8-year-old boy with anemia, high-grade fever, back pain, cough and limping was referred to our hospital. Due to cough and limping, which was suspiciously related to the right knee joint tenderness, CXR and right knee joint anteroposterior (AP) X-ray were taken. Perihilar and retrocardiac infiltration is noticed grossly in CXR; however, bone density seems reduced with an abnormal lower thoracic and upper lumbar vertebral shape (Figure 6A). Thoracolumbar AP and lateral (Lat) views were requested and again multiple collapsed vertebrae were seen (Figure 6B and C). Metaphyseal translucency with reduced bone density was noted in the distal femoral metaphysis (Figure 6D). Bone marrow aspiration confirmed ALL.

### 2.7. Case 7

A 6-year-old girl with pallor and a few weeks of right elbow pain was admitted. During evaluation, mild tenderness over both knee joints with mild swelling and mild degree fever were found. X-rays of the right forearm and knee joint revealed periosteal reaction in the proximal radial metaphysis. Other abnormalities were thickened metaphyseal translucency in the right distal forearm bones, bilateral distal femurs and proximal tibiae, which was more prominent in the latter (Figure 7). Nearby bone density is also reduced. This case was ALL confirmed by bone marrow aspiration.

### 2.8. Case 8

A 9-year-old boy was admitted to our hospital with mild groin pain, which became worst in the recent days. The patient had limping with more severely affected left hip joint. Pelvic X-ray revealed reduced height of femoral epiphysis with osteochondral fracture on the left side due to avascular necrosis. There is also permeative appearance in the proximal femoral metaphyses bilaterally in this new ALL case proven by bone marrow aspiration and CBC (Figure 8).

### 2.9. Case 9

A 13-year-old girl with chief complaints of fever, pallor, abdominal pain, headache, bone pain and weight loss was admitted. High-grade fever and cachexia were found on examination. Tenderness on touching extremities and skull were noticed. On abdominal examination, mild organomegaly was detected; however, no significant tenderness could be seen. The patient was sent for bone survey X-ray. Multiple lytic lesions on skull X-ray (Figure 9A), permeative bony lesions in iliac bones, reduced vertebral height of L5 with reduced bone density and a lucent zone beneath the vertebral endplate was detected (Figure 9B and C). Reduced bone density with permeative (small lytic lesions) in the proximal tibial bone adjacent to knee joints were more obvious (Figure 9D). However, frank abnormality in bilateral scapular bones in CXR was noted as pearmeative lytic lesions. Perihilar-peribronchial infiltration was also present (Figure 9E). This case proved to be ALL.

### 2.10. Case 10

A 2-year-old girl presented with fever and shortness of breath. CXR was taken in the emergency department (Figure 10A). Widening of the anterior mediastinum and right side pleura is noticeable. Double contour appearance of thymus on the left side suggestive of infiltration of thymus with increasing suspicion of leukemia was seen. Similar to case 5, there was metaphyseal erosion in the left humeral medial metaphysis better shown in Figure 10B. CBC and bone marrow aspiration finally confirmed ALL.

## 3. Discussion

The commonest malignancy in children is acute leukemia, which may be found primarily by the bony lesions [2][3][7][11][12]. Bone pain due to proliferation of bone marrow, pressure effect, compress fractures and osteoporosis may ensue [3][7][13].

Bony lesions are more common in leukemic cases [9][14][15]. One of the most important radiological findings is metaphyseal lucent band, which could be seen in 10-48% of the cases [9][16]. Osteopenia may be seen in various degrees depending on socioeconomical conditions and poor general conditions before illness. For example, the rate was 22% in Muller’s [9], 9% in Sinigaglia’s [15], 62.5% in Kobayashi’s [17] and 14% in Jeong’s [12] study. Osteosclerotic lesions, which are considered as bone marrow infarct or may be due to leukemic cell infiltration in the bone marrow may be seen in 7.4%-31% of the cases [2][9][10][15][16]. Compress vertebral bone fracture has been reported variously up to 31.25% in different articles [12][14][17].

The skull has been an uncommon site of involvement; however, periosteal reaction and osteolytic lesions have been reported at various ranges by different investigators 4-11% for periosteal reaction and 13-23% for osteolytic lesions [2][9][10][16][17].

It seems that more frequent bone lesions in younger age may be due to a more active bone marrow at this age.

Bone involvement is more common among leukemia in children so familiarity with these bony lesions helps the radiologist to consult other physicians more accurately for quick reference and accurate diagnosis, so the bone survey should be done in every case suspicious of having leukemia.
